# Nursing leadership strategies in addressing COVID-19 in light of John Kotter’s framework

**DOI:** 10.1590/0034-7167-2023-0289

**Published:** 2024-06-28

**Authors:** Patrícia Alves Galhardo Varanda, Gilberto Tadeu Reis da Silva, Simone Coelho Amestoy, Vânia Marli Schubert Backes, Gabriela Marcellino de Melo Lanzoni, Augusto Ferreira Umpiérrez

**Affiliations:** I Universidade Federal da Bahia. Salvador, Bahia, Brazil; II Universidade Federal do Vale do São Francisco. Petrolina, Pernambuco, Brazil; III Universidade Federal de Santa Catarina. Florianópolis, Santa Catarina, Brazil; IV Universidad Católica del Uruguay. Montevideo, Uruguai

**Keywords:** Leadership, Nurses, Nursing, COVID-19, Change Management, Liderazgo, Enfermeras y Enfermeros, Enfermería, COVID-19, Gestión del Cambio

## Abstract

**Objective:**

To analyze the leadership strategies of nurses in university hospitals in response to care management changes during the COVID-19 pandemic, informed by John Kotter’s insights.

**Methods:**

This multicentric study utilized qualitative and analytical methods. It was conducted through semi-structured interviews with 139 lead nurses from 10 university hospitals in Brazil. Data analysis included Bardin’s content analysis and the webQDA software.

**Results:**

The primary category identified was “Nursing Leadership Strategies in the Battle Against COVID-19,” encompassing five subcategories. This category underscored the importance of strategic vision in nursing leadership for combating COVID-19 within hospital settings, as well as the necessity of working collaboratively with their teams and other healthcare professionals.

**Final Considerations:**

The results highlight the strategies used by lead nurses in confronting COVID-19, which can be associated with John Kotter’s theoretical framework and his model of change.

## RESUMEN

**Objetivo:**

analizar las estrategias de liderazgo de enfermeros en hospitales universitarios frente a los cambios en la gestión del cuidado en el enfrentamiento de la COVID-19 a la luz de John Kotter.

**Métodos:**

investigación multicéntrica, de tipo cualitativa y analítica. Se realizó mediante entrevistas semiestructuradas, con 139 enfermeros líderes, de 10 hospitales universitarios, en Brasil. En el análisis de datos se utilizó el análisis de contenido de Bardin y el software webQDA.

**Resultados:**

se obtuvo una categoría principal “Estrategias de liderazgo de enfermería en el enfrentamiento de la COVID-19”, y cinco subcategorías. Destacando el liderazgo de los enfermeros por la importancia de la visión estratégica para enfrentar la COVID-19 dentro de las instituciones hospitalarias, así como la necesidad de trabajar conjuntamente con sus equipos y otros profesionales de la salud.

**Consideraciones Finales:**

los resultados evidenciaron las estrategias utilizadas por los enfermeros líderes en el enfrentamiento de la COVID-19, siendo posible relacionarlas con el marco teórico de John Kotter y su modelo de cambio.

## INTRODUCTION

In different healthcare settings, the nurse’s role as a leader, coordinator, or team manager is evident. Nursing accounts for more than half of the workforce in the health sector, and in recognition of its significant role, the World Health Organization (WHO), the International Council of Nurses (ICN), and the All-Party Parliamentary Group on Global Health (APPG) launched the Nursing Now campaign to strengthen the role of nursing^(1)^. However, during the Nursing Now campaign, the spread of COVID-19 began, which originated in Wuhan, China, in December 2019, and became a serious public health problem, peaking from 2020 to 2022. Thus, in 2020, the WHO declared a pandemic situation^(2-3)^.

In this context, the SARS-CoV-2 virus (severe acute respiratory syndrome coronavirus)^(4)^ caused health problems that affected work processes and demanded leadership to implement necessary changes and make appropriate decisions. During the COVID-19 pandemic, nurses’ leadership stood out in facing challenges such as the lack of human and material resources^(5-6)^. Therefore, it was essential for lead nurses to become skilled and seek strategies to guide team members^(5-6)^. Nurses were at the forefront of the fight against COVID-19 and are essential in healthcare.

Given this, it is crucial for lead nurses to present new strategies for the (re)organization of nursing in various dimensions, whether emotional, physical, political, social, or economic^(7)^, especially in scenarios of unexpected changes. Leaders need to be planners and strategists, ensuring an adequate direction towards the desired goal with efficacy. It is necessary to communicate the vision of what will be achieved, in addition to developing skills to ensure successful planning^(8-9)^.

In the meantime, this study is based on the theoretical framework of John Kotter, an internationally recognized author in leadership and change, who created the “eight-step change process” model, enabling the understanding and management of changes through leaders. Kotter asserts that one cannot ignore the daily demands of an organization; it is necessary to identify risks and opportunities in advance, devising creative strategic actions with enough agility to implement them quickly^(10)^.

Despite all the issues generated in global health, it is worth mentioning that there were no strategic plans to deal with the COVID-19 pandemic. Therefore, the WHO, the Brazilian Ministry of Health, the Centers for Disease Control and Prevention (CDC, USA), and other national and international health institutions suggested the application of contingency plans for influenza, due to the clinical and epidemiological similarities with respiratory viruses^(11)^.

Strategic plans were not readily available, emphasizing the need for leadership from nurses and other health professionals to develop coping strategies imposed by the challenges of COVID-19^(11-12)^. Essential is nursing leadership that focuses on care management, seeking new strategies that encompass the implementation of new actions, protocols, and restructuring to face the disease caused by SARS-CoV2^(12)^.

Therefore, it is crucial to discuss leadership in nursing to contribute to care and quality in assistance. Thus, the study adopts John Kotter’s concepts as a theoretical reference, due to his esteemed work “Leading Change,” in which he describes his model and contributes to helping leaders prevent mistakes and execute changes effectively^(13)^.

The “eight-step change process” model comprises: establishing a sense of urgency (awareness of the necessity for organizational change); forming a guiding coalition (creating a group with the authority to lead the change); developing vision and strategy (crafting an appropriate, simple, and clear vision to facilitate major changes, motivating people, and coordinating strategies towards a future vision); communication (conveying the vision to achieve the common goal of change); empowering (enhancing people’s abilities); achieving short-term wins (acknowledging efforts towards transformation); consolidating gains (balancing change actions for integration into the organizational culture); and embedding new approaches in the culture (continuously solidifying change)^(13-14)^.

In this context, understanding the eight-step change process model can aid leaders in various organizations, such as lead nurses in health institutions. This involves providing a step-by-step guide on effectively exercising and facilitating intrinsic organizational change, particularly during crises like the COVID-19 pandemic.

Therefore, this research is warranted due to the absence of a national and Latin American perspective that addresses these concepts in the COVID-19 context and that presents the eight-step change process model as a tool to aid leaders, notably lead nurses.

Consequently, this research is framed by the following guiding question: What are the leadership strategies of nurses in university hospitals regarding changes in care management when confronting COVID-19, as seen through John Kotter’s lens?

## OBJECTIVE

To analyze the leadership strategies of nurses in response to changes in care management during the COVID-19 pandemic, in accordance with John Kotter’s theories.

## METHODS

### Ethical Aspects

This study is part of a broader multicentric project on changes in nursing care management in a hospital setting. The original project received approval from the Research Ethics Committee of the reference institution, as well as from the Research Ethics Committees of all participating institutions, encompassing 10 University Hospitals in Brazil. The Informed Consent Form (ICF) was made available online on a research explanation page, allowing participants to express their consent to the study by clicking an icon labeled “I agree to participate in the research.” Some interviews were also conducted in person, where the FICF was presented to the participants. Both in-person and online formats were adopted due to the interviews taking place during the COVID-19 pandemic, and the in-person researchers were already vaccinated against the coronavirus.

Notably, the interviews were recorded using codes representing the educational institutions affiliated with these hospitals, such as UFBA 01, UFPA 01, UFRJ 01, following a sequential numerical order. Moreover, compliance with Resolutions No. 466/2012 and 510/2016 of the National Health Council^(15-17)^ was meticulously observed.

### Study Type

This study is a multicentric qualitative and analytical research, forming part of the overarching project “Evaluation of Nursing Care for COVID-19 Patients in Brazilian University Hospitals.” The Consolidated Criteria for Reporting Qualitative Research (COREQ) were utilized to structure this study.

### Study Setting

The research was conducted in 10 Brazilian Federal University hospitals, which are recognized as medium and high complexity reference centers for the Unified Health System (SUS in portuguese)^(15)^. Eight of these hospitals are affiliated with the Brazilian Hospital Services Company (EBSERH). Geographically, the hospitals are situated in the following Brazilian regions: two in the South region, including the parent institution; two in the Southeast region; two in the Northeast region, where the proposing institution is located; two in the North region; and two in the Central-West region. The selection of these locations aimed to explore varied realities and strategies in addressing COVID-19.

### Data Source

The study included 139 nurses working across various sectors of the health institutions, involved in patient care for COVID-19, and who met the inclusion criteria of at least three months of experience with these patients. Participants were informed about the criteria at the time of invitation to the study, which was conducted either online or in person, taking into account the limitations imposed by the coronavirus pandemic and the goal of reaching a broader participant base.

### Data Collection and Organization

Data collection occurred between March 2021 and April 2022, utilizing Google Forms® and in-person interviews conducted by researchers from the respective educational institutions. The study’s data corresponds to the semi-structured interviews carried out, beginning with a socio-professional characterization of the participants, encompassing aspects like age, gender, sector of activity, duration of service to COVID-19 patients, professional role, and years of experience.

In the second stage, open-ended questions were posed to align with the objectives of the parent project and to facilitate discussion on the changes, leadership, and management strategies perceived by nurses during the pandemic. Prior to the commencement of data collection across all institutions, a pilot test was undertaken with four nurses to evaluate the applicability of the questions, accompanied by online meetings with all participating researchers to deliberate and refine the questions, ensuring alignment with the objectives of the parent project and related studies.

### Data Analysis

For data analysis, Bardin’s Content Analysis method was utilized. The process began with a “floating reading” as part of the pre-analysis phase, focusing on organizing the collected material and enabling the researcher to read and re-read the content for hypothesis formulation. The second phase involved a more intensive reading of the content, leading to the creation of categories. At this stage, the researcher employed Microsoft Word® to help separate topics and define potential categories. The third phase entailed processing the results, where the researcher checked if the findings aligned with the proposed objectives and performed decoding.

For this phase, the webQDA software was selected, enhancing qualitative research by facilitating secure and collaborative online data analysis by other researchers. It is crucial to note that the software does not conduct any analysis independently but assists in organizing, editing, and visualizing the identified categories and subcategories. Consequently, the primary category of this research was identified: nursing leadership strategies in addressing COVID-19, comprising five subcategories: strategic vision in care; agility in decision-making; adoption of new technologies; communication tactics and skills; and strategies for managing emotions.

## RESULTS

Regarding socio-professional characterization, the 139 nurses worked in various sectors of the health institutions, but all attended to COVID-19 patients. Their ages ranged from 23 to 65 years, with an average age of 32. Of these, the majority were female (n=122; 87.77%) and male (n=17; 12.23%). As for professional experience, it varied from one to 36 years, and in terms of academic qualifications, four had doctoral degrees and 36 had master’s degrees.

The 139 responses were analyzed using Bardin’s Content Analysis method and with the aid of the webQDA software. This resulted in the primary category: Nursing Leadership Strategies in Addressing COVID-19 and its five subcategories, described below.

### Nursing Leadership Strategies in Addressing COVID-19

In this primary category, lead nurses highlighted the importance of strategy in combating COVID-19 within their hospital institutions, as well as the need to work together with their teams and other healthcare professionals to create effective strategies. This led to the identification of five subcategories: strategic vision in care; agility in decision-making; adoption of new technologies; communication tactics and skills; and strategies for managing emotions. The nurse’s leadership was emphasized as an essential element in transforming these strategies into applied and targeted actions.

### Strategic Vision in Care

In this subcategory, the testimonies of the lead nurses emphasize the importance of the third stage of Kotter’s change model, Vision and Strategy. They stress that a leader needs to have a broad vision beyond COVID-19, recognizing that care involves various elements and that clarity is needed to create a set of effective strategies.

*Managing care means having a broad vision* [...] *seeing beyond the patient’s illness.* [...] *I have to look beyond COVID. The patient is on spontaneous ventilation, lucid and oriented, with some emotional demand* [...] *So to manage care is to broaden your vision, to go beyond the illness, to really see that the patient is a whole* [...]. (UFBA 01)

*The leadership of a Nurse is to coordinate, to observe, to do and to teach, and above all, to coordinate,* [...] *one must have the vision of an eagle, must see everything, because the coordinator is very responsible so they must have a grand vision of what they are doing, they must know what they are doing, where they are, and where they want to go, because if they do not have this vision they will get lost along the way* [...]. (UFPA 09)

*Regarding the organization of care* [...] *as it was a new disease I think no one was prepared* [...] *the issue of staffing, there were some different things that we did not account for* [...] *for example, the time spent donning and doffing PPE*.[...] *there would need to be an improvement in the issue of these resources to ensure higher quality care* [...] *because when we do not have the appropriate material* [...] *our assistance is also compromised* [...]. (UNIFESP 03)

### Agility in Decision-Making

The second subcategory highlights the need for agility in decision-making, such as increasing the number of beds and trained personnel, as well as restructuring the physical space in hospitals. The lead nurses demonstrated agility in making decisions to face these challenges and in training the nursing team for the care of this unknown disease at the time. Reflecting Kotter’s model, the testimonies embody stages such as a Sense of Urgency and Strategic Vision.

*From my point of view, management was both a breakthrough and an avalanche* [...] *imagine going from coordinating 56 professionals to 160... today, in the ICU I have 160 professionals because I went from an ICU of 10 beds to one with 31 beds*. [...] *in relation to the COVID patient, it’s really that we need both the structural apparatus that we had, as well as the apparatus of professionals qualified to work with the critical patient* [...]. (UFAM 02)

*The selection process was based on professionals with vast experience, professionals who worked at another job. So, those who came to us were handpicked, very experienced and they helped us immensely.* [...] *We had ICU sector head nurses who had a very good vision of care* [...]. (UFMS 02)

[...] *newly hired staff through an emergency selection process and a great challenge began* [...] *a large number of people who were not trained in the institution’s routine* [...] *truly a war scenario but, in terms of leadership, no colleague complained, we were able to carry out all services, I managed to carry out the nursing process* [...]. (UFSC 02)

[...] *it was also distressing to be closed in, because the Covid ICU was set up in the most isolated ICU of the hospital* [...] *with the goal of trying to isolate from the rest of the hospital, because we did not want to contaminate the rest of the patients - which was a correct idea - and to have ambulance access,* [...] *so as not to have to move this patient through the inside of the hospital* [...]. (UFMS 01)

Another issue was the decision-making by lead nurses to address the shortage of PPEs. Although the first strategy was not always the most appropriate, the nurses continued to seek solutions and also showed the importance of nursing in assuming multiple tasks during the pandemic.

*Initially, there was difficulty due to the fear of running out of PPE, so the nursing team practically performed all roles, from transporting for tests to delivering food* [...] *in contact with the management today we have managed to greatly reduce this workload.* (UFSC 02)

*In the beginning, it was difficult; we never ran out, but supplies were limited and use was rationed, so you couldn’t be changing masks all the time. The PFF2, N95 masks were more allocated to the ICU and medical clinic professionals* [...]. (UFMT 06)

*So, the issue of PPEs was difficult because the pharmacy didn’t want to release the amount, and in the COVID-19 sector, there is a high demand for PPE, a high demand for PPE.* [...] *There were difficulties related to the linen supply as well* [...] *Thus, the interaction with other services was complicated* [...] *there started to be a bureaucrat* [...] *a person who provides support* [...] *since we could not be leaving the sector* [...]. (UFRN 04)

### Adoption of New Technologies

The third subcategory underscores the importance of technological innovation as a strategy for dealing with COVID-19, which has proven efficient and improved care. The relationship with Kotter’s model is particularly evident in the third stage, Strategic Vision, which motivates the team to make the change happen, and even in Kotter’s final stage, where the transformation becomes part of the institutional culture. It also relates to the sixth stage of Kotter’s model, achieving short-term wins, as these technologies improved the work process.

*All the bureaucratic and administrative parts did not exist during COVID; we changed everything to online. So, today, most things have become online.* [...] (UFBA 8)

*Video calls, the cell phone that we had inside was used at the time of the visit for the family outside to call. Families received this number before the visit began and as I passed the cell phone around, they would call, and they could also call at other times.* (UFMS 01)

*The main technology in care management* [...] *were the video calls, through a tablet.* [...] *Our electronic medical record system, within the nursing care systematization, has a form of a care plan checklist which helps us a lot.* [...] (UFMT 02)

[...] *I remember that we tried to give a lot of guidance to people, visitors, companions, we even made a booklet on how this reception of the baby, the isolation, would be conducted, how the medical visit would be passed on, which could not be in person, everything by phone was made into a booklet, an explanatory leaflet.* (UFMT 01)

[...] *But we now had innovations that were added in the field of physiotherapy. The COVID patient in physiotherapy, for instance, as he generates aerosols, we included in our contingency plan that he had to have a specific filter, which is the “HEPA”* [...]. (UFAM 02)

### Communication Tactics and Skills

In the fourth subcategory, the importance of the leader’s communication skills is highlighted, relating to the fourth stage of Kotter’s change model. The nurses’ testimonies reinforce the need for clear, respectful, and secure communication of visions and strategies, using adapted communication tools like discussion circles. It is evident that it is fundamental for nurses to develop this skill.

*The main strategies are: Secure Communication, which we call Closed-loop communication, secure communication; another strategy is to develop skills that are not just technical skills but also relationship skills* [...] *that will enable you to lead well* [...] *to make communication, to strengthen your support network in the place where you work.* (UFBA 1)

[...] *one thing that we used a lot was the conversation circle, right there on the floor, to define some flows, some care, some priorities; so it was the tool we used the most: the conversation circle, right there on the floor.* (UFSM 03)

[...] *It is always necessary to establish good communication with team members, whether in relation to care protocols, how they are made, and the training. These trainings that were carried out in the initial planning phase are intended to guide, while subsequent trainings and audits are necessary to measure the effectiveness of treatments. It is not enough just to train, it is important to know the team’s adherence to that training* [...]. (UFRJ 01)

### Strategies for Dealing with Emotions

This final subcategory signals the significance of emotional understanding and support for healthcare professionals on the COVID-19 frontline. Nurses acknowledge the emotional impact of the pandemic on themselves, their colleagues, and patients’ families and strive to provide emotional support and create strategies to mitigate these effects. Emotional sensitivity is seen as a crucial factor for successful change, aligning with Kotter’s idea that the essence of change is in emotions. Leaders need to see and feel the change, motivate the team, and empower them.

*And there was another thing that had a significant impact, which was the emotional aspect of the families, because the patient did not receive visits. So we created the ‘window visit’ project* [...] *we had glass windows and then right at the beginning of the pandemic* [...] *When the pandemic started and it was announced “there will be no visits”, it was distressing* [...] *I spoke with the social worker, with the occupational therapist: “What if we bring the family to see the patient here through the window?”. And it worked.* [...] (UFMS 01)

*We receive patients who are very anxious, afraid of imminent death, constantly asking us to help them not to die* [...] *So the psychological, emotional part, it’s a part that impacts* [...] *for me this is something that causes me difficulty in my management* [...]. (UFRJ 12)

*I think there is a lack of psychological support, socio-functional follow-up, I am even making contact with a dedicated service for this, to try to seek alternatives* [...] *Mental health is a very vast field and has specialties, so I am realizing that my nurses are needing this support* [...]. (UFRJ 18)

*The greatest difficulty was the psychological aspect, the fear of having a family member in there with us, afraid of receiving friends, afraid of needing a bed, or needing a bed for a family member, and being inside, knowing how the situation was, and knowing that there would not be* [beds][...]. (UFMS 01)

## DISCUSSION

Our data confirm Kotter’s principles and reveal the leadership strategies necessary to address COVID-19 in Brazilian university hospitals. The primary category that emerged was ‘Nursing Leadership Strategies in Addressing COVID-19,’ which encompassed five subcategories.

The first subcategory emphasized the strategy used by lead nurses and highlighted their strategic vision in care. They recognized the importance of adopting a comprehensive approach in managing nursing care and the need to transcend the disease caused by COVID-19. Nurses assumed multiple roles, such as coordination, education, and training of the nursing staff, which requires a clear vision to prevent deviations.

In this sense, the vision and strategy from Kotter’s change model suggest that, in the best cases, leaders refine clear, simple, and sensible visions that motivate and inspire a set of strategies. In less successful situations, strategies demonstrate slowness and caution, potentially resulting in leadership failures in a rapidly evolving scenario^(18)^.

The COVID-19 pandemic introduced a set of activities that needed to be planned and organized to mitigate the contagion of the virus. In the role of a manager, the nurse’s leadership was essential to ensure best practices in structural reorganization, reduction of infection rates, patient safety, and a demonstration of their skills in managing people and communicating with the team and patients^(19)^.

Therefore, the importance of a clear and comprehensive vision regarding strategies is reinforced, so they can be effectively applied. Kotter emphasizes that with an appropriate vision, people understand the reasons they should fight, feeling motivated and encouraged to undertake the necessary actions for change^(14,18)^. Notably, during COVID-19, lead nurses and their teams understood the true reason for the changes, and their strategies were fundamental in achieving the desired outcomes.

In the second subcategory, the strategies of lead nurses are distinguished by their agility in decision-making regarding the sudden increase in the number of beds, staff training, and improvements in physical restructuring. These were various challenges that needed to be addressed quickly, as nurses had to deal with a significant increase in professionals assisting COVID-19 patients overnight, leading to an increased number of beds and even an internal restructuring of the health institution. Some hospitals succeeded in hiring qualified professionals, while others struggled to find staff prepared to treat critical COVID-19 patients, especially in intensive care units.

Hence, revisiting Kotter’s concepts, we can associate them with some stages of his change model, such as the Sense of Urgency and Vision and Strategy. The author indicates that significant changes start with creating a Sense of Urgency; it is necessary for people to see and feel the urgency, thus recognizing the need for transformations. To achieve this, obtaining the necessary cooperation is crucial^(14,18)^.

In this context, hospitals required preparation to expand their care in a planned and organized manner. Thus, health managers created strategic plans, such as treatment protocols, contingency plans, and training strategies for health professionals, for the care of COVID-19 patients^(20)^. A restructuring of health institutions became necessary, involving interconnections between care management, changes in work organization, strengthening of human resources, supplies, and technologies. This restructuring led to increased visibility of nursing practice, requiring nurses to be capable of developing new strategies to ensure patient safety^(21)^.

Also, in the second subcategory, the need for quick decision-making became apparent in response to challenges related to PPE that nursing faced during the pandemic. The scarcity or lack of PPE significantly impacted nursing care, prompting nurses to assume additional responsibilities, such as conducting laboratory tests, delivering meals to patients, and dealing with reduced supplies from the pharmacy and laundry sectors.

A study^(21)^ conducted in Brazil highlighted the issue of PPE, indicating that the lack of this protective equipment contributed to occupational risk exposure and was challenging for nursing professionals at the pandemic’s onset. The Federal Nursing Council (COFEN) recorded that more than 44,308 nursing professionals were infected, and over 467 died. The study underscored the effectiveness of PPE in protecting against the virus and stressed that hospitals must ensure an adequate supply of PPE to workers. Moreover, nursing leaders need to create strategies for the proper use and training of the health team^(21)^.

Thus, nursing stood at the forefront of addressing another sense of urgency stemming from COVID-19. The lead nurse, confronted with numerous issues, played a pivotal role, and their actions for change were crucial to ensuring the reduction of contagion and the quality of care, demonstrating their valuable vision for the future.

Regarding the third subcategory, the strategy of nurses was revealed through technological innovations, showing that university hospitals resorted to certain technologies, such as online medical records, video calls via cell phones or tablets (for patients’ families), and online informational leaflets. Consequently, lead nurses displayed improvements in care management, and these improvements can be associated with the short-term wins of Kotter’s change model.

A study in the United Kingdom confirmed that the coronavirus disease altered the dynamics of health care globally. The study emphasized that a significant change at a large hospital in the UK was the implementation of telemedicine. To carry out this transformation, they employed Kotter’s eight-step model, which resulted in the model providing efficiency in implementation and agility in the process^(22)^.

In the fourth subcategory, the strategies highlighted by lead nurses centered on communication. They emphasized secure communication, the importance of a support network, the use of discussion circles to navigate new workflows, and recognized that establishing strong communication with the team was invaluable throughout the entire process of change brought on by COVID-19.

Kotter’s model includes a stage that underscores the importance of a leader’s communication. Leaders who fail to communicate effectively may send inconsistent messages14. According to his model, following the development of a vision and strategy, the next step is communicating the direction of change to the team. This message should be clearly and widely disseminated throughout the institution to ensure it is well understood^(18)^.

A study from Australia delved into communication in the context of COVID-19 and suggested that communication strategies should take precedence to help mitigate the risk of emerging diseases and bolster efforts to control their spread. It highlighted that recognizing and communicating the problem is the first step toward implementing solutions^(23)^. Similarly, a study in Portugal that examined the theme of communication in nursing during the COVID-19 crisis emphasized the significance of this skill in nursing. It valued effective communication with patients as a vital tool for improving care. The study also expressed concerns about communication challenges due to PPE, which hindered understanding among team members and sometimes made it difficult to hear another professional^(24)^.

Therefore, it is crucial for the lead nurse to develop communication skills, given their role in mediating individual relationships, particularly in a work setting that demands respectful and trustful team interactions to collectively achieve the set goals.

In the final category, lead nurses describe strategies directed at emotional perceptions. At one research site, lead nurses proposed the ‘window visit’ project, allowing family members to see their hospitalized loved ones since visits were restricted during the COVID-19 situation.

The pandemic gave rise to various fears, such as the fear of infecting family and friends, the fear of infection, and the fear of losing loved ones and patients. Some lead nurses noted the absence of a robust psychological support strategy for health professionals. Consequently, COVID-19 profoundly impacted the emotional well-being of not only nurses but all health care workers.

A study conducted at a Brazilian university hospital found that nursing professionals are subject to psychological strain, high stress, and even the onset of depression, adversely affecting job satisfaction and the quality of care delivered. In the COVID-19 context, the mental health of nursing professionals was particularly affected by the challenge of confronting the unknown, leading to signs of anxiety and depression among workers^(25)^.

In his book ‘The Heart of Change,’ Kotter posits that individuals are less likely to be transformed by analysis than by having a clear vision of the truth, allowing them to see and feel; in other words, change is rooted in people’s emotions^(18)^.

The pandemic has intensely affected people’s emotions, with each person confronting their fears daily in the struggle to overcome them-for themselves, their families, friends, and patients. Lead nurses, at the forefront of caring for patients with COVID-19, devised numerous essential strategies to alleviate the chaos wrought by the coronavirus. The sense of ‘empowerment’ played a key role in motivating professionals to utilize their problem-solving abilities, resilience, and in solidifying gains through small but meaningful actions that made a significant impact.

In this context, Kotter’s eight-step model of change is an important leadership tool that can aid lead nurses in guiding the process of leading changes within health organizations, aimed at improving the quality of care. It contributes to the consolidation of nursing strategies and continuous improvements in health care. This model is established based on a sense of urgency that raises awareness about the true purpose of transformations, through key individuals with the authority to direct the changes and who ensure a clear vision and strategies to influence people to achieve the purpose of the change^(10,14)^.

### Study Limitations

The study faced limitations, including difficulty in securing a larger participant base, as data collection occurred during the delicate phases of the COVID-19 pandemic. Participants, often burdened by their workload, sometimes canceled scheduled interviews. Furthermore, in the locations where interviews were conducted, a high number of other COVID-19 studies were underway, leading to participant exhaustion and, consequently, some declining to participate.

### Contributions to the Field of Nursing

COVID-19 introduced numerous challenges and uncertainties that impacted global health, healthcare institutions, and their health professionals. These professionals had to rapidly develop strategies for leadership and management to address and restructure, which directly influenced the role of the lead nurse, increasing their responsibilities. This study aims to contribute to discussions on the strategic actions of lead nurses in the pandemic scenario, offering a new perspective based on Kotter’s change model. It seeks to assist other nursing leaders in their practice, especially in crisis situations where leadership plays a critical role. The study emphasizes the importance of documenting and sharing the experiences of lead nurses on the front lines of COVID-19. Well-trained and informed nursing leaders, aligned with new evidence, can contribute to improving nursing care.

## FINAL CONSIDERATIONS

We identified significant leadership strategies executed by nurses in Brazilian university hospitals, which were relatable to the precepts and some stages of John Kotter’s change model. Among the strategies, we highlighted valuable elements for executing effective leadership, such as the importance of a strategic vision, strategies in response to PPE shortages, technological innovations, improved communication methods, and emotional perceptions. Leadership skills need to be developed by nurses to ensure they are prepared and strengthened in their practice in both everyday and crisis situations. It is essential for healthcare institutions to contribute to and promote training in nursing leadership, empowering these professionals and ensuring the quality of care.
