# Supplementary material for: Nursing leadership strategies in addressing COVID-19 in light of John Kotter’s framework
Source: Rev Bras Enferm. 2024 Jun 28;77(Suppl 1):e20230289. doi: 10.1590/0034-7167-2023-0289 (PMC11213536; doi:10.1590/0034-7167-2023-0289)
Supplement: Translation

# Translation: Estratégias de liderança de enfermeiros no enfrentamento da covid-19 à luz de John Kotter

**DOI:** 10.1590/0034-7167-2023-0289pt

**Authors:** Patrícia Alves Galhardo Varanda, Gilberto Tadeu Reis da Silva, Simone Coelho Amestoy, Vânia Marli Schubert Backes, Gabriela Marcellino de Melo Lanzoni, Augusto Ferreira Umpiérrez

**Affiliations:** I Universidade Federal da Bahia. Salvador, Bahia, Brasil; II Universidade Federal do Vale do São Francisco. Petrolina, Pernambuco, Brasil; III Universidade Federal de Santa Catarina. Florianópolis, Santa Catarina, Brasil; IV Universidad Católica del Uruguay. Montevideo, Uruguai

**Keywords:** Liderança, Enfermeiras e Enfermeiros, Enfermagem, covid-19, Gestão de Mudança

## Abstract

**Objetivo:**

analisar as estratégias de liderança de enfermeiros em hospitais universitários diante das mudanças na gestão do cuidado no enfrentamento da covid-19 à luz de John Kotter.

**Métodos:**

pesquisa multicêntrica, do tipo qualitativa e analítica. Ocorreu por meio de entrevistas-semiestruturadas, com 139 enfermeiros-líderes, de 10 hospitais universitários, no Brasil. Na análise de dados utilizou a análise de conteúdo de Bardin e o software *webQDA*.

**Resultados:**

obteve-se uma principal categoria “Estratégias de liderança de enfermeiros no enfrentamento da covid-19”, e cinco subcategorias. Destacando a liderança de enfermeiros pela importância da visão estratégica para enfrentar a covid-19 no interior das instituições hospitalares, bem como a necessidade de trabalhar em conjunto com suas equipes e demais profissionais de saúde.

**Considerações Finais:**

os resultados evidenciaram as estratégias utilizadas por enfermeiros-líderes no enfrentamento da covid-19, sendo possível de relacionar com o referencial teórico de John Kotter e seu modelo de mudança.

## INTRODUÇÃO

Nos diferentes contextos dos serviços de saúde, evidencia-se o protagonismo do enfermeiro enquanto líder, coordenador ou gestor de equipe. A enfermagem representa mais da metade da força de trabalho na área da saúde e, em reconhecimento ao seu relevante papel, a Organização Mundial de Saúde (OMS), o Conselho Internacional de Enfermeiros (CIE) e o *All-Party Parliamentary Group on Global Health* (APPG) criaram a campanha *Nursing Now* para fortalecer o papel da enfermagem^(1)^. Contudo, em plena campanha *Nursing Now*, iniciou-se a disseminação da covid-19, que surgiu em Wuhan, China, em dezembro de 2019, e se tornou um grave problema de saúde pública com ápice nos anos de 2020 a 2022. Sendo assim, no ano de 2020, a OMS declarou situação de pandemia^(2-3)^.

Nesse sentido, o vírus SARS-CoV-2 (do inglês: *severe acute respiratory syndrome coronavirus*)^(4)^ ocasionou problemas de saúde que afetaram os processos de trabalho e demandaram liderança para implementar as mudanças necessárias e tomar decisões adequadas. Durante a pandemia da covid-19, a liderança dos enfermeiros se destacou diante dos desafios enfrentados, como a falta de recursos humanos e materiais^(5-6)^. Deste modo, foi essencial que os enfermeiros-líderes se capacitassem e buscassem estratégias para conduzir os membros da equipe^(5-6)^. Enfermeiros estiveram na linha de frente da luta contra a covid-19 e são essenciais no cuidado da saúde.

Diante disso, é fundamental que os enfermeiros-líderes apresentem novas estratégias para a (re)organização da enfermagem em diversas dimensões, sejam elas: emocional, física, política, social ou econômica^(7)^, em especial em cenários de mudanças inesperadas. Os líderes precisam ser planejadores e estrategistas, assegurando uma adequada condução ao objetivo desejado com eficácia. É necessário comunicar a visão do que será realizado, além de desenvolver habilidades para garantir que o planejamento ocorra com sucesso^(8-9)^.

Nesse ínterim, este estudo se baseia no referencial teórico de John Kotter, autor reconhecido internacionalmente no âmbito de liderança e mudanças e que criou o modelo do “processo de mudança de oito etapas”, possibilitando compreender e gerenciar mudanças por meio de líderes. Kotter afirma que não se pode ignorar as demandas diárias de uma organização; é necessário identificar os riscos e as oportunidades com antecedência, elaborando ações estratégicas criativas e com agilidade suficiente para inseri-las rapidamente^(10)^.

Apesar de toda a problemática gerada na saúde global, cabe mencionar que não havia planos estratégicos para lidar com a pandemia da covid-19. Então, a OMS, o Ministério da Saúde do Brasil, o *Centers for Disease Control and Prevention* (CDC, Estados Unidos), entre outras instituições nacionais e internacionais de saúde, sugeriram a aplicação de planos de contingência para influenza, por causa das semelhanças clínicas e epidemiológicas com os vírus respiratórios^(11)^.

Os planos estratégicos não estavam prontamente disponíveis, enfatizando a necessidade de liderança de enfermeiros e outros profissionais de saúde para desenvolverem estratégias de enfrentamento impostas pelos desafios da covid-19^(11-12)^. É essencial uma liderança de enfermagem que se preocupe com a gestão do cuidado, buscando novas estratégias que englobem a implementação de novas ações, protocolos e se reestruturem para enfrentar essa doença ocasionada pelo SARS-CoV2^(12)^.

Para tanto, considera-se crucial a importância de se discutir a liderança na enfermagem para contribuir com o cuidado e a qualidade na assistência. Assim, o estudo adota os conceitos de John Kotter como referencial teórico, devido à sua conceituada obra “*Leading Change*” (Liderando Mudanças), na qual descreve seu modelo e contribui para que os líderes previnam erros e consigam executar mudanças com eficácia^(13)^.

O modelo de “processo de mudança de oito etapas” consiste em: estabelecer um senso de urgência (consciência da necessidade de organização para a mudança); formação de uma coalizão administrativa (criação de um grupo com autoridade para liderar a mudança); desenvolvimento de visão e estratégia (criação de uma visão adequada, simples e clara para facilitar grandes mudanças, motivando as pessoas e coordenando as estratégias, por meio de um propósito claro direcionado para uma visão de futuro); comunicação (comunicar a visão para alcançar o objetivo comum da mudança); empowerment (fortalecer a capacitação das pessoas); conquistas de curto prazo (valorizar os esforços para a transformação); consolidação de ganhos (alcançar um equilíbrio nas ações de mudança para serem absorvidos na cultura organizacional); e instituição de novos métodos na cultura (consolidação da mudança de forma contínua)^(13-14)^.

Nesse sentido, compreender o modelo de processo de mudança de oito etapas pode auxiliar líderes em diferentes tipos de organizações, como é o caso dos enfermeiros-líderes em instituições de saúde. Assim, elucidar um passo a passo sobre como exercer e facilitar o processo de mudança intrínseco das organizações de forma eficaz, principalmente em momentos de crises, como foi durante a pandemia da covid-19.

Portanto, essa pesquisa se justifica devido à falta de um panorama nacional e latino-americano que aborde esses conceitos no contexto da covid-19 e que tragam o modelo de processo de mudança de oito etapas como uma ferramenta que possibilite auxiliar os líderes, em especial, os enfermeiros-líderes.

Dessa forma, delineou-se esta pesquisa com a seguinte questão norteadora: quais são as estratégias de liderança de enfermeiros em hospitais universitários diante das mudanças na gestão do cuidado no enfrentamento da covid-19 à luz de John Kotter?

## OBJETIVO

Analisar as estratégias de liderança de enfermeiros diante das mudanças na gestão do cuidado no enfrentamento da covid-19, à luz das teorias de John Kotter.

## MÉTODOS

### Aspectos éticos

Este estudo integra um macroprojeto multicêntrico sobre mudanças na gestão do cuidado de enfermeiros no contexto hospitalar. Obteve-se aprovação do projeto original pelo Comitê de Ética em Pesquisa da instituição de referência e também pelas aprovações dos Comitês de Ética em Pesquisa de todas as instituições proponentes participantes, totalizando 10 Hospitais Universitários no Brasil. O Termo de Consentimento Livre e Esclarecido (TCLE) foi disponibilizado online em uma página de esclarecimento sobre a pesquisa, permitindo que os participantes confirmassem sua anuência ao estudo clicando em um ícone com a opção “concordo em participar da pesquisa”. Algumas entrevistas presenciais também foram realizadas, nas quais o TCLE foi lido aos participantes. Optou-se pelos dois formatos, presencial e online, devido às entrevistas ocorrerem durante a pandemia de covid-19, e os pesquisadores que participaram presencialmente já haviam sido vacinados contra o coronavírus. É válido sinalizar que as entrevistas foram salvas em códigos os quais representam as instituições de ensino que esses hospitais estão vinculados como, por exemplo, UFBA 01, UFPA 01, UFRJ 01 e respeitando uma ordem numérica sequencial. Contudo, buscou-se atender às Resoluções nº 466/2012 e 510/2016 do Conselho Nacional de Saúde^(15-17)^.

### Tipo de estudo

Trata-se de uma pesquisa multicêntrica de natureza qualitativa, do tipo analítica, vinculada ao projeto matriz “Avaliação do Cuidado de Enfermagem a Pacientes com covid-19 em Hospitais Universitários Brasileiros”. Utilizou-se o instrumento *Consolidated Criteria for Reporting Qualitative Research* (COREQ) para estruturar este estudo.

### Cenário do estudo

A pesquisa foi realizada em 10 hospitais universitários federais brasileiros, considerados referência de média e alta complexidade para o Sistema Único de Saúde (SUS)^(15)^, sendo oito deles vinculados à Empresa Brasileira de Serviços Hospitalares (EBSERH). Geograficamente, os hospitais estão distribuídos nas seguintes regiões brasileiras: dois na região Sul, incluindo a instituição matriz; dois na região Sudeste; dois na região Nordeste, onde se localiza a instituição proponente; dois na região Norte; e dois na região Centro-Oeste. A escolha desses locais visou explorar as diferentes realidades e estratégias de enfrentamento da covid-19.

### Fonte de dados

O estudo contou com a participação de 139 enfermeiros atuantes em diferentes setores das instituições de saúde, envolvidos na assistência ao paciente com covid-19 e que atendiam ao critério de inclusão de no mínimo três meses de atuação com esses pacientes. Os participantes foram informados dos critérios no momento do convite para o estudo, de forma online ou presencial, tendo em vista as limitações impostas pela pandemia do coronavírus e o intuito de alcançar um maior número de participantes.

### Coleta e organização dos dados

A coleta de dados ocorreu entre março de 2021 e abril de 2022, por meio do *Google Forms*® e de entrevistas presenciais conduzidas pelos pesquisadores das instituições de ensino. Os dados desta pesquisa correspondem às entrevistas semi-estruturadas realizadas, que incluíram uma primeira etapa de caracterização socioprofissional dos participantes, abordando aspectos como idade, sexo, setor de atuação, tempo de atendimento a pacientes com covid-19, função e experiência profissional em anos.

Na segunda etapa, foram feitas perguntas abertas com o objetivo de atender aos objetivos do projeto matriz e discutir as mudanças e estratégias de liderança e gestão percebidas pelos enfermeiros durante a pandemia. Antes do início da coleta de dados em todas as instituições, foi realizado um teste piloto com quatro enfermeiros para avaliar as questões a serem aplicadas, bem como reuniões online com todos os pesquisadores envolvidos para discutir e ajustar as perguntas de modo a responder aos objetivos do projeto matriz e demais projetos vinculados.

### Análise dos dados

Para a análise dos dados, utilizou-se o método de Análise de Conteúdo de Bardin. Inicialmente, realizou-se uma “leitura flutuante” como parte da pré-análise, etapa dedicada à organização do material coletado e à leitura e releitura pelo pesquisador para a elaboração das hipóteses. Na segunda etapa, procedeu-se a uma leitura mais intensa do conteúdo, criando as categorias. Neste momento, o pesquisador utilizou o programa *Microsoft Word*® como auxílio para separar os assuntos e definir as possíveis categorias. A terceira etapa consistiu no tratamento dos resultados, momento em que o pesquisador revisou se os achados estavam atendendo aos objetivos propostos e realizou a decodificação. Para esta fase, optou-se pela utilização do software *webQDA*, que fortalece as pesquisas qualitativas ao permitir que outros pesquisadores analisem os dados de forma segura e colaborativa online. É importante ressaltar que o software não realiza nenhum tipo de análise por conta própria, mas facilita na organização, edição e visualização das categorias e subcategorias emergidas. Assim, foi possível evidenciar a categoria principal dessa pesquisa: estratégias de liderança de enfermeiros no enfrentamento da covid-19, e suas cinco subcategorias: visão estratégica no cuidado; agilidade na tomada de decisões; adoção de novas tecnologias; táticas e habilidades de comunicação; estratégias para lidar com o emocional.

## RESULTADOS

Quanto à caracterização socioprofissional, os 139 enfermeiros atuaram em diferentes setores das instituições de saúde, mas todos atenderam pacientes com covid-19. A idade variou de 23 a 65 anos, com uma média de 32 anos. Desses, a maioria era do sexo feminino (n=122; 87,77%) e masculino (n=17; 12,23%). Quanto ao tempo de experiência profissional, este variou de um a 36 anos, e no que se refere ao nível de titulação, quatro possuíam doutorado e 36 tinham mestrado.

As 139 respostas foram analisadas por meio do método de Análise de Conteúdo de Bardin e com o auxílio do *software webQDA*. Isso resultou na principal categoria: Estratégias de liderança de enfermeiros no enfrentamento da covid-19 e suas cinco subcategorias, descritas abaixo.

### Estratégias de liderança de enfermeiros no enfrentamento da covid-19

Nessa principal categoria, os enfermeiros-líderes destacaram a importância da estratégia para enfrentar a covid-19 em suas instituições hospitalares, bem como a necessidade de trabalhar em conjunto com suas equipes e demais profissionais de saúde para criarem estratégias eficazes. Isso levou à identificação de cinco subcategorias: visão estratégica no cuidado; agilidade na tomada de decisões; adoção de novas tecnologias; táticas e habilidades de comunicação; estratégias para lidar com o emocional. A liderança do enfermeiro foi destacada como um elemento essencial para transformar essas estratégias em ações aplicadas e direcionadas.

### Visão estratégica no cuidado

Nesta subcategoria, os depoimentos dos enfermeiros-líderes ressaltam a importância da terceira etapa do modelo de mudança de Kotter, a Visão e Estratégia. Eles enfatizam que o líder necessita ter uma visão ampla, além da covid-19, reconhecendo que o cuidado envolve vários elementos e que é preciso ter clareza para criar um conjunto de estratégias eficazes.

*Gerir cuidados é você ter uma visão ampla* [...] *ver além da doença do paciente.* [...] *Eu tenho que ver além da covid. Ele está em ventilação espontânea, ele está lúcido e orientado, ele tem alguma demanda emocional* [...] *então você gerir o cuidado é você ampliar a visão é você ir além da doença é você, realmente vê que o paciente é um conjunto* [...]. (UFBA 01)

*A liderança do Enfermeiro é coordenar, é observar, é fazer e ensinar e, principalmente, coordenar,* [...] *tem que ter a visão de águia, tem que ver tudo, porque o coordenador ele é muito responsável então ele tem que ter uma visão grande daquilo que ele está fazendo, ele tem que saber o que ele está fazendo, de onde ele está pra onde ele quer chegar, porque se ele não tiver essa visão ele vai se perder no caminho* [...]. (UFPA 09)

*Em relação à organização do cuidado* [...] *por ser uma doença nova acho que ninguém estava preparado* [...] *a questão de dimensionamento de pessoal, teve algumas coisas diferentes que a gente não contabilizava* [...] *por exemplo, tempo gasto de paramentação e desparamentação.* [...] *precisaria ter uma melhoria mesmo na questão desses recursos pra poder garantir o cuidado de maior qualidade* [...] *porque quando a gente não tem o material adequado* [...] *a nossa assistência também fica prejudicada* [...]. (UNIFESP 03)

### Agilidade na tomada de decisões

Na segunda subcategoria, emerge a necessidade de agilidade na tomada de decisões, como aumentar o número de leitos e de pessoal capacitado, bem como reestruturar o espaço físico nos hospitais. Os enfermeiros-líderes demonstraram ser ágeis ao tomar decisões para enfrentar esses desafios e capacitar a equipe de enfermagem para o cuidado desta desconhecida doença na época. Trazendo para o modelo de Kotter, os depoimentos refletem etapas como o Senso de Urgência e Visão Estratégica.

*No meu ponto de vista, na gestão foi tanto um avanço quanto uma avalanche* [...] *imagina eu sair da coordenação de 56 profissionais para 160... hoje, na UTI eu tenho 160 profissionais, porque eu saí de uma UTI de 10 leitos para uma de 31 leitos.* [...] *em relação ao paciente covid, é que realmente a gente precisa de um aparato tanto estrutural que nós tínhamos, tanto quanto o aparato de profissionais habilitados para trabalhar com o paciente crítico* [...]. (UFAM 02)

*O processo de seleção foi feito em cima de profissionais já com vasta experiência, profissionais que trabalhavam em outro emprego. Então, essas pessoas que vieram para a gente foram pessoas escolhidas a dedo, com muita experiência e nos ajudou demais.* [...] *Nós tivemos enfermeiras chefes de setores de UTI, que tinham uma visão muito boa de atendimento* [...]. (UFMS 02)

[...] *colaboradores recém-contratados por um processo seletivo emergencial e começou um grande desafio* [...] *grande número de pessoas e que não eram treinadas quanto a rotina da instituição* [...] *realmente um cenário de guerra mas, assim, a parte de liderança os colegas nenhum reclamaram, a gente conseguia executar todo serviço, consegui* (eu) *fazer o processo de enfermagem* [...]. (UFSC 02)

[...] *foi angustiante também a parte de ficar fechado, porque a UTI covid foi feita na UTI que era mais isolada do hospital* [...] *com o objetivo de tentar isolar do restante do hospital, porque não queria se contaminar o resto dos pacientes - o que foi uma ideia acertada - e de ter o acesso de ambulância,* [...] *para não precisar passar com esse paciente por dentro do hospital* [...]. (UFMS 01)

Outra questão foi a tomada de decisões pelos enfermeiros-líderes para enfrentar a escassez de EPIs. Embora nem sempre a primeira estratégia tenha sido a mais adequada, os enfermeiros continuaram buscando soluções e mostrando também a importância da enfermagem em assumir múltiplas tarefas durante a pandemia.

[...] *dificuldade inicialmente pelo receio de faltar EPIs, então praticamente a equipe de enfermagem ela desempenhava todos os papéis, transporte para exames, entregar alimentação* [...] *em contato com a gestão hoje em dia já conseguimos diminuir muito mais essa carga de trabalho.* (UFSC 02)

[...] *de início foi difícil, nunca chegou a faltar, mais era reduzido e o uso era racionado, então não podia ficar trocando máscara toda hora, as máscaras PFF2, N95 eram mais destinados aos profissionais da UTI e da clínica médica* [...]. (UFMT 06)

[...] *então tanto a questão dos EPI’s era difícil, porque a farmácia não queria liberar a quantidade, e EPI no setor covid-19 é uma grande demanda de EPI, tem uma grande demanda de EPI.* [...] *Tinha dificuldade em relação a rouparia também* [...] *então, a interação com os outros serviços era complicada.* [...] *começou a ter um burocrata* [...] *uma pessoa que dá apoio* [...] *já que a gente não poderia ficar saindo do setor* [...]. (UFRN 04)

### Adoção de novas tecnologia

A terceira subcategoria evidencia a importância da inovação tecnológica como uma estratégia para lidar com a covid-19, que se mostrou eficiente e melhorou o cuidado. A relação com o modelo de Kotter é percebida, principalmente na terceira etapa, a Visão Estratégica, que motiva a equipe para que a mudança aconteça, e até mesmo na última etapa de Kotter, em que a transformação entra na cultura institucional. Relaciona-se também com a sexta etapa do modelo de Kotter, as conquistas de curto prazo, uma vez que essas tecnologias melhoraram o processo de trabalho.

[...] *toda a parte burocrática e administrativa não existia na época do covid* (sic)*, a gente modificou tudo para o online. Então, tudo hoje, a maioria das coisas, se tornou tudo online*. [...] (UFBA 8)

*Videochamada, esse celular que a gente tinha lá dentro era usado na hora da visita para família lá de fora ligar. As famílias recebiam esse número antes da visita começar e aí conforme eu ia passando o celular elas iam ligando e elas podiam ligar também em outros momentos.* (UFMS 01)

*A principal tecnologia no gerenciamento do cuidado* [...] *foram as videochamadas, através de um tablet.* [...] *O nosso prontuário eletrônico, ele possui no sistema da sistematização da assistência de enfermagem, uma forma de um checklist do nosso plano de cuidados que nos ajuda bastante.* [...]. (UFMT 02)

[...] *eu lembro que a gente tentar fazer bastante orientações para as pessoas, as pessoas que vinham visitar, os acompanhantes, foi feito até uma cartilha de como que era essa recepção do bebê, ali do isolamento, como que seria passada essa visita médica, que não poderia ser pessoalmente, tudo por telefone foi feito uma cartilha, um folheto explicativo.* (UFMT 01)

[...] *Mas tínhamos agora inovações que foram adicionadas no campo da fisioterapia. O paciente covid em fisioterapia, ele não pode, por exemplo, como ele gera aerossóis, colocamos no nosso plano de contingência que ele tinha que ter um filtro específico, que é o “EPA”* [...]. (UFAM 02)

### Táticas e habilidades de Comunicação

Na quarta subcategoria, destaca-se a importância da habilidade de comunicação do líder, relacionada à quarta etapa do modelo de mudanças de Kotter. Os depoimentos dos enfermeiros reforçam a necessidade de uma comunicação clara, respeitosa e segura das visões e estratégias, utilizando ferramentas de comunicação adaptadas, como rodas de conversa. É evidenciado que é fundamental para o enfermeiro desenvolver essa habilidade.

*As principais estratégias são: Comunicação Segura, que a gente chama de Comunicação em alça fechada comunicação segura, outra estratégia é você desenvolver habilidades que não são habilidades técnicas apenas desenvolver habilidades de relacionamento* [...] *que vão fazer com que você consiga liderar bem aquilo* [...] *fazer comunicação você fazer fortalecer a sua rede de apoio no lugar onde você trabalha.* (UFBA 1)

[...] *uma coisa que a gente utilizou bastante foi a roda de conversa, na ilha mesmo então, para definir alguns fluxos, alguns cuidados, algumas prioridades; então foi a ferramenta que a gente mais utilizou: foi a roda de conversa, mesmo, na ilha.* (UFSM 03)

[...] *Sempre é necessário estabelecer boa comunicação com os membros da equipe, seja em relação aos protocolos assistenciais, como eles são feitos e os treinamentos. Esses treinamentos que foram realizados na fase inicial do planejamento possuem o intuito de orientar, já os treinamentos e auditorias subsequentes são necessários para que se possa medir a efetividade dos tratamentos. Não adianta apenas treinar, é importante saber qual é a adesão da equipe a aquele treinamento* [...]. (UFRJ 01)

### Estratégias para lidar com o emocional

Esta última subcategoria sinaliza a significância da compreensão emocional e do apoio aos profissionais de saúde na linha de frente da covid-19. Os enfermeiros reconhecem o impacto emocional da pandemia sobre si mesmos, seus colegas e familiares dos pacientes e se esforçam para fornecer apoio emocional e criar estratégias para mitigar esses efeitos. A sensibilidade emocional é vista como um fator crucial para uma mudança bem-sucedida, alinhando-se com a ideia de Kotter de que o cerne da mudança está nas emoções. Os líderes precisam ver e sentir a mudança, motivar a equipe e empoderar.

*E teve mais uma coisa que pegou bastante, que foi o aspecto emocional das famílias, porque o paciente não recebia visita. E aí nós criamos o projeto da visita na janela* [...] *então nós tínhamos janelas de vidro e aí logo no começo da pandemia* [...] *Então, quando começou a pandemia e falou “não vai ter visita”, isso foi angustiante* [...] *falei com a assistente social, com a terapeuta ocupacional: “Ah! E se a gente trouxer a família pra ver o paciente aqui pela janela?”. E deu certo.* [...] (UFMS 01)

[...] *A gente recebe pacientes muito ansiosos, com medo de morte iminente, a todo momento pedindo para a gente ajudá-los a não morrer* [...] *então a parte psicológica, emocional, ela é uma parte que impacta* [...] *para mim isso é uma coisa que me causa uma dificuldade na minha gestão* [...]. (UFRJ 12)

*Eu acho que falta um pouco de assistência psicológica, um acompanhamento sócio-funcional, inclusive eu estou fazendo contato com um serviço próprio disso, para tentar buscar alternativas* [...] *Saúde mental é um campo muito vasto e tem especialidade, então eu estou percebendo que os meus enfermeiros estão precisando desse apoio* [...]. (UFRJ 18)

*A maior dificuldade foi a questão psicológica, o medo de ter algum familiar lá dentro conosco, com medo de receber amigos, medo de ter que precisar de um leito, ou precisar de um leito para um familiar, e estar lá dentro, sabendo como estava a situação, e saber que não iria ter* [leitos] [...]. (UFMS 01)

## DISCUSSÃO

Nossos dados confirmam os princípios de Kotter e revelam as estratégias de liderança necessárias para enfrentar a covid-19 nos hospitais universitários brasileiros. A principal categoria que emergiu foi ‘Estratégias de liderança de enfermeiros no enfrentamento da covid-19’, que contou com cinco subcategorias.

A primeira subcategoria enfatizou a estratégia utilizada pelos enfermeiros líderes e evidenciou a visão estratégica desses profissionais frente ao cuidado. Assim, eles reconheceram a importância de adotar uma abordagem abrangente na gestão do cuidado de enfermagem e a necessidade de transcender a doença por covid-19. Os enfermeiros assumiram vários papéis, como a coordenação, educação e capacitação da equipe de enfermagem, o que requer uma visão clara para evitar desvios.

Nesse sentido, a visão e estratégia, do modelo de mudança de Kotter, sinalizam que, nos melhores casos, os líderes aprimoram visões nítidas, simples e sensatas, as quais motivam e inspiram um conjunto de estratégias. Em situações menos bem-sucedidas, as estratégias demonstram lentidão e precaução, o que pode resultar em falhas por parte dos líderes em um cenário em rápida evolução^(18)^.

A pandemia da covid-19 trouxe um conjunto de atividades que necessitaram ser planejadas e organizadas para amenizar o contágio do vírus. No que concerne ao enfermeiro, seu papel de gestor e sua liderança foram essenciais para assegurar: melhores práticas na reorganização estrutural, redução nos índices de infecção, segurança do paciente e destaque de sua habilidade na gestão de pessoas e comunicação com a equipe e pacientes^(19)^.

Desta forma, reforça-se a importância de uma visão clara e abrangente diante das estratégias para que possam ser aplicadas com eficácia. Kotter reforça que, em uma visão adequada, as pessoas conseguem compreender a razão pela qual devem lutar, sentindo-se motivadas e encorajadas para atender às ações necessárias da mudança^(14,18)^. Nota-se que, na covid-19, os enfermeiros-líderes e sua equipe sabiam o verdadeiro motivo para as mudanças, e suas estratégias foram fundamentais para alcançar os objetivos almejados.

Na segunda subcategoria, as estratégias dos enfermeiros líderes destacam-se pela agilidade frente à tomada de decisões acerca do aumento repentino no número de leitos, capacitações dos funcionários e melhorias na reestruturação física. Trata-se de diferentes desafios que necessitaram ser solucionados com celeridade, pois praticamente da noite para o dia os enfermeiros tiveram que lidar com um aumento considerável de profissionais na assistência ao paciente com covid-19, consequentemente, aumento no número de leitos e até mesmo uma reestruturação interna da instituição de saúde. Alguns hospitais tiveram sucesso na contratação de profissionais qualificados, enquanto outros tiveram dificuldades em encontrar pessoal preparado para atender pacientes críticos com covid-19, especialmente para unidades de terapia intensiva.

Destarte, voltando aos conceitos de Kotter, conseguimos relacionar com algumas etapas do seu modelo de mudança, como, por exemplo, o Senso de Urgência e a Visão e Estratégia. O autor sinaliza que mudanças expressivas começam com a elaboração do Senso de Urgência; é necessário que as pessoas possam ver e sentir a urgência, assim percebem que precisam realizar transformações. Para tanto, é crucial obter a cooperação necessária^(14,18)^.

Nesse contexto, os hospitais demandaram um preparo na ampliação acerca da assistência de maneira planejada e organizada, e, assim, os gestores de saúde criaram planos estratégicos, como protocolos de atendimento, planos de contingência e estratégias de capacitações para os profissionais de saúde, para a assistência ao paciente com covid-19^(20)^. Uma reestruturação nas instituições de saúde se fez necessária, envolvendo interconexões entre a gestão do cuidado, mudanças na organização do trabalho, fortalecimento dos recursos humanos, insumos e tecnologias. Isso ocasionou uma visibilidade da prática do enfermeiro, necessitando que estes estivessem aptos para o desenvolvimento de novas estratégias de cuidado, assegurando a segurança do paciente^(21)^.

Ainda na segunda subcategoria, foram necessárias rápidas tomadas de decisão diante dos desafios relacionados aos EPIs que a enfermagem enfrentou durante a pandemia. A escassez ou falta de EPIs teve um impacto significativo na assistência de enfermagem, e os enfermeiros tiveram que assumir responsabilidades adicionais, como realizar exames laboratoriais, entregar refeições aos pacientes e lidar com suprimentos reduzidos dos setores de farmácia e lavanderia.

Um estudo^(21)^, realizado no Brasil, também evidenciou essa questão dos EPIs, sinalizando que a ausência desse equipamento de proteção contribui para a exposição a riscos ocupacionais, e foi desafiador para os profissionais da enfermagem logo no início da pandemia. O Conselho Federal de Enfermagem (COFEN) registrou que mais de 44.308 profissionais de enfermagem foram infectados, e mais de 467 foram a óbito. O estudo enfatizou a eficácia do EPI na proteção contra o vírus e que os hospitais devem garantir o fornecimento adequado de EPIs aos trabalhadores, e os líderes de enfermagem necessitam criar estratégias para o uso adequado e treinamento da equipe de saúde^(21)^.

Isto posto, a enfermagem esteve à frente de mais um dos sensos de urgência oriundos da covid-19. O enfermeiro-líder, diante de tantas problemáticas, teve seu protagonismo, e suas ações para a mudança foram essenciais para assegurar a redução de contágio e a qualidade na assistência, evidenciando sua visão de futuro como valiosa.

Frente à terceira subcategoria, a estratégia dos enfermeiros se revela diante das inovações tecnológicas, emergindo que os hospitais universitários recorreram a algumas tecnologias, como prontuários em formato on-line, vídeo-chamadas por meio de celulares ou tablets (para os familiares dos pacientes) e folhetos informativos via on-line. Desta forma, os enfermeiros-líderes evidenciaram melhorias na gestão do cuidado, e essas melhorias podemos relacioná-las com as conquistas de curto prazo do modelo de mudança de Kotter.

Um estudo, no Reino Unido, reforçou que a doença pelo coronavírus mudou a dinâmica da assistência em saúde em todo o mundo. O estudo destacou que a mudança realizada em um hospital de grande porte, no Reino Unido, foi a implementação da telemedicina e, para implementarem essa transformação, utilizaram o modelo de oito etapas de Kotter, o qual resultou que o modelo trouxe eficácia na implementação e agilidade no processo^(22)^.

Na quarta subcategoria, as estratégias destacadas pelos enfermeiros-líderes foram acerca da comunicação. Os enfermeiros-líderes destacaram uma comunicação segura, garantindo uma rede de apoio, rodas de conversas frente aos novos fluxos, e o estabelecimento de uma boa comunicação com a equipe foi valioso diante de todo o processo de mudança enfrentado pela covid-19.

Kotter, por sua vez, traz em seu modelo uma etapa descrita com a importância da comunicação de um líder. Líderes que não se comunicam direito podem passar mensagens inconsistentes^(14)^. Em seu modelo, após a visão e estratégia, o próximo passo é a comunicação para a equipe, assim, a direção da mudança deve ser amplamente comunicada e difundida em toda a instituição de forma que a mensagem fique bem compreendida^(18)^.

Na Austrália, um estudo trouxe discussões acerca da comunicação no contexto da covid-19 e menciona que estratégias de comunicação devem vir em primeiro plano para contribuir com o risco de surgimento de novas doenças e aumentar os esforços para controlar a sua disseminação. Mostrando que reconhecer e comunicar o problema é o primeiro passo para a implementação de soluções^(23)^. Um estudo, em Portugal, buscou abordar a temática da comunicação em enfermagem no cenário da covid-19 e apontou a importância desta competência para a enfermagem, valorizou a comunicação efetiva com o paciente, sendo essa uma ferramenta efetiva para melhorias dos cuidados. Apresentou também preocupações de comunicação por causa dos EPIs que dificultavam o entendimento da comunicação entre a equipe, ou por dificuldade até mesmo de conseguir ouvir o outro profissional^(24)^.

A comunicação, portanto, necessita ser desenvolvida pelo enfermeiro-líder, devido à sua importância em ser a mediadora entre as relações dos indivíduos, principalmente, no âmbito do trabalho que exige uma interação respeitosa e de confiança entre a equipe, para que consigam alcançar juntos o objetivo proposto.

A última categoria, os enfermeiros-líderes descrevem as estratégias voltadas para as percepções emocionais. Em um dos locais da pesquisa, enfermeiros-líderes criaram a proposta do projeto ‘visita na janela’, para que os familiares pudessem ver seu ente querido, já que no cenário da covid-19 as visitas eram proibidas.

Essa pandemia desencadeou várias formas de medo, como medo de infectar familiares e amigos, medo de se infectar, medo de perder entes queridos e pacientes. Alguns enfermeiros líderes relataram a falta de uma estratégia mais robusta de apoio psicológico aos profissionais de saúde. Portanto, a covid-19 afetou profundamente o bem-estar emocional não apenas dos enfermeiros, mas também de todos os profissionais de saúde.

Um estudo^(25)^, realizado em um hospital universitário brasileiro, revela que no trabalho de enfermagem o profissional está exposto a desgastes psicológicos, estresse elevado e até mesmo ao desencadeamento de depressão. Isso interfere negativamente na satisfação com o trabalho e na qualidade do cuidado ofertado. No contexto da covid-19, a saúde mental dos profissionais de enfermagem foi mais impactada pelo desafio de lidar com o desconhecido, resultando em sinais de ansiedade e depressão entre os trabalhadores^(25)^.

Em sua obra ‘O coração da mudança’, Kotter sinaliza que as pessoas se transformam menos baseadas em análises, mas saem da sua zona de conforto ao terem uma visão clara da verdade, ao conseguirem ver e sentir, ou seja, a mudança está nas emoções das pessoas^(18)^.

Esta pandemia afetou intensamente as emoções das pessoas, cada uma vivenciando seus medos e lutando a cada dia contra eles para vencê-los, por si mesmas, pela família, amigos e pacientes. Destacando-se os enfermeiros-líderes, que estavam na linha de frente da assistência ao paciente com covid-19, eles criaram tantas estratégias essenciais para amenizar todo o caos gerado pelo coronavírus. O sentimento de *‘empowerment*’ foi importante para mobilizar os profissionais na sua capacidade de resolução, resiliência e consolidação de ganhos, em pequenos, mas valiosos gestos que fizeram a diferença.

Nessa conjuntura, o modelo de oito etapas de mudança de Kotter é um instrumento importante de liderança que pode contribuir com o enfermeiro-líder no direcionamento acerca do processo de liderar mudanças no interior das organizações de saúde, voltado para a qualidade da assistência. Assim, contribui com a consolidação de estratégias de enfermagem e melhorias contínuas no cuidado em saúde. Devido a este ser estabelecido a partir de um senso de urgência que conscientiza sobre o verdadeiro objetivo das transformações, por meio de pessoas-chave com autoridade para direcionar as mudanças e, que assegurem uma visão e estratégias claras para influenciar pessoas a alcançarem o propósito da mudança^(10,14)^.

### Limitações do estudo

As limitações do estudo referem na dificuldade de adesão maior de participantes, devido a coleta de dados ocorrer em momentos ainda delicados da pandemia pela covid-19. Por questões próprias de sobrecarga de trabalho, e mesmo com agendamento prévio alguns participantes cancelavam o agendamento. Nos lócus das entrevistas também ocorriam um número elevado de outras pesquisas sobre a covid-19, e alguns participantes encontravam-se exauridos, assim, declinando a participação.

### Contribuições para a área da enfermagem

A covid-19 trouxe inúmeros desafios e incertezas que impactaram a saúde global, as instituições e seus profissionais de saúde. Estes necessitaram encontrar rápidas estratégias acerca da liderança e gestão para o enfrentamento e reestruturação, o que implicou diretamente no papel do enfermeiro-líder, aumentando suas responsabilidades.

Desse modo, este estudo buscou contribuir com discussões sobre as ações estratégicas do enfermeiro-líder no cenário pandêmico, trazendo um novo olhar baseado no modelo de mudança de Kotter. Visa auxiliar outros líderes de enfermagem em sua prática, especialmente em situações de crise nas quais a liderança desempenha um papel crítico. O estudo enfatiza a importância de documentar e compartilhar as experiências dos enfermeiros líderes na linha de frente da covid-19. Líderes de enfermagem bem capacitados e informados, alinhados com novas evidências, podem contribuir para melhorar os cuidados de enfermagem.

## CONSIDERAÇÕES FINAIS

Identificamos relevantes estratégias de liderança executadas pelos enfermeiros dos hospitais universitários brasileiros, as quais foram possíveis de relacionar com os preceitos e algumas etapas do modelo de mudança de John Kotter. Dentre as estratégias, foi possível destacar elementos valiosos para a execução de uma boa liderança, como a importância de uma visão estratégica, estratégias diante da escassez de EPIs, inovações tecnológicas, melhores formas de comunicação e percepções emocionais.

A competência de liderança necessita ser desenvolvida pelos enfermeiros, para que, em situações cotidianas e de crise, estejam aptos e fortalecidos em sua prática na assistência ao cuidado. É fundamental que as instituições de saúde contribuam e promovam capacitações acerca da liderança dos enfermeiros, promovendo o empoderamento desses profissionais e assegurando a qualidade dos cuidados.

Os achados deste estudo evidenciam os esforços dos enfermeiros-líderes na criação e execução de estratégias, as quais envolvem uma reestruturação dos processos de mudança no contexto hospitalar, colaborando com a qualidade na assistência e amenizando os impactos oriundos da pandemia.
